# Prognostic Significance and Clinicopathological Correlations of Epigenetic MGMT Gene Silencing in High Grade Diffuse Gliomas

**DOI:** 10.15190/d.2023.14

**Published:** 2023-09-26

**Authors:** Alka Singh, Anurag Singh, Sarita Agrawal, Awadhesh Jaiswal, Sushila Jaiswal

**Affiliations:** ^1^Department of Pathology, Sanjay Gandhi Post Graduate Institute of Medical Sciences Lucknow, India; ^2^Department of Medical Genetics, Sanjay Gandhi Post Graduate Institute of Medical Sciences, Lucknow, India; ^3^Department of Neurosurgery, Sanjay Gandhi Post Graduate Institute of Medical Sciences, Lucknow, India; ^4^Department of Pathology, Sanjay Gandhi Post Graduate Institute of Medical Sciences, Lucknow, India

**Keywords:** Alkylating agent, ATRX, Glioblastoma, IDH1, MGMT promoter methylation, TP53, temozolomide.

## Abstract

Glioblastoma is the most aggressive and commonest primary malignant brain tumour. Current standard of care includes surgery, radiation, and alkylating agent chemotherapy. Despite multimodal treatment, the survival of glioblastoma patients is dismal. Loss of O6-methylguanine-DNA-methyltransferase(MGMT) protein expression due to promoter methylation reduces glioma cell DNA repair activity and resistance to alkylating agents. Thus, in world health organization (WHO) grade 4 diffuse glioma patients treated with an alkylating agent, methylated MGMT promoter is currently being considered a clinically relevant prognostic as well as predictive biomarker. Our aim was to assess the frequency of MGMT promoter methylation in WHO grade 4 diffuse glioma patients and study their prognostic role and clinicopathological correlations. A two-year prospective cohort research was conducted on 89 WHO grade 4 diffuse glioma patients. The clinical and demographic data were retrieved from our hospital information system. MGMT methylation was assessed using methylation specific polymerase chain reaction. Data was analysed using SPSS-24 software. We studied 89 cases of WHO grade 4 diffuse glioma, of which 38.2% showed methylation of MGMT promoter. There was no significant difference in age, sex, location of tumor and clinical presentation between the methylated and unmethylated groups. A statistically significant association of methylated MGMT promoter was observed with isocitrate dehydrogenase-1 (IDH1) protein expression (p = 0.050) and alpha-thalassemia/mental retardation syndrome X-linked (ATRX) loss (p = 0.003). No significant association was noted with p53 overexpression (p = 0.492) and Ki-67 index (p = 0.698). The median overall survival in these patients receiving standard radiotherapy and concomitant temozolomide chemotherapy showed a trend towards better survival in group with methylated MGMT promoter (p < 0.001). Our study suggests that methylation of MGMT promoter is more frequent in the subset of grade 4 diffuse gliomas that significantly exhibit IDH1 immunopositivity and loss of ATRX expression. Also, patients who receive radiation therapy and simultaneous temozolomide chemotherapy have a considerably better prognosis and treatment outcome, if the promoter region of MGMT is methylated.

## Introduction

Glioblastoma (WHO grade 4) is the most aggressive and commonest of all primary malignant brain tumors. The annual incidence is ~3–4 per 100,000 population and is associated with poor prognosis^1^. Assessment of the primary tumor specimens on histology and evaluation of isocitrate dehydrogenase 1/2 (IDH1/2) status on immunohistochemistry (IHC) is currently the gold standard method of diagnosis for all WHO grade 4 diffuse gliomas^2^. In glioblastoma, the survival of patients is dismal despite aggressive treatment protocol, counting for approximately 15-17 months^3^. The treatment protocol currently followed includes surgical resection of primary tumor, followed by radiation therapy, and six cycles of chemotherapy with an alkylating agent like temozolomide (TMZ)^4^. The cellular toxicity of alkylating agents is exerted through alkylation of guanine at its O6 position^5^. O6-methylguanine-DNA-methyltransferase (MGMT), a DNA repair enzyme, rapidly eliminates these alkyl adducts over guanine, and thus counteracts the cytotoxic effect exerted by alkylating agents by preventing the formation of cross-links^6^. This causes resistance to temozolomide^7^. Methylation of MGMT promoter region leads to loss of expression of MGMT protein causing a reduction in the DNA repair activity of glioma cells and thus prevents their resistance to temozolomide^8-10^. The outcome of glioblastoma patients treated with temozolomide is therefore inversely related to the level of expression of enzyme MGMT. Hypermethylation of MGMT promoter highly regulates the expression of MGMT protein and provokes transcriptional silencing^11^. Thus, in WHO grade 4 diffuse glioma patients treated with temozolomide, methylated MGMT promoter is now being considered an important and clinically relevant prognostic as well as predictive biomarker^12,13^.

Pyrosequencing and methylation-specific polymerase chain reaction (MS PCR) are among the two most popular methods, currently in use to evaluate the methylation status of MGMT promoter region. MS PCR is a qualitative technique and utilise both methylation and unmethylation- specific primers. These primers amplify separately fully methylated and unmethylated sequences of MGMT promoter respectively^14^. There are a few studies in literature that have identified methylated MGMT promoter as an independent prognostic factor in WHO grade 4 diffuse glioma patients. Hence, evaluation of the methylation status of MGMT promoter region is currently considered mandatory for selection of patients in clinical trials^13,15-17^.

Aim of our study was to determine the frequency of MGMT promoter methylation in a cohort of WHO grade 4 diffuse glioma patients and correlate the findings with various clinical, pathological and immunohistochemical markers expression, specifically IDH1, tumor protein 53 (TP53), alpha-thalassemia/mental retardation syndrome X-linked (ATRX), and Ki-67 proliferation index. We have also tried to provide a brief comparison of the findings in our study with those available in the literature.

## Methods

A prospective cohort study was conducted in the Departments of Pathology and Neurosurgery at a tertiary care referral centre for two years, where 89 consecutive cases of WHO grade 4 diffuse gliomas, were included. Patient’s demographic and clinical details were retrieved from our hospital information system (HIS) and medical record files. The data included: patient’s age at the time of diagnosis, presenting signs and symptoms, site of localization of tumor, imaging findings, extent of surgical resection, type and timing of adjuvant therapy, follow-up time and overall survival in months. Details of the treatment were obtained from all the patients. All these patients were treated with radiation therapy and concomitant chemotherapy with alkylating agent temozolomide (dose: 75 mg/m^2^ body surface area daily) after surgical excision of their primary tumor. The tissue retrieved after neurosurgical tumor resection was processed and further analysed by an experienced neuropathologist. Immunohistochemistry was also carried out in all these cases.

Immunohistochemical procedures

Immunohistochemistry was performed using standard antigen retrieval methods on a 4-μm-thick formalin fixed paraffin embedded tissue sections. The conventional streptavidin biotin peroxidase immunohistochemistry approach was used to evaluate the expression of IDH1 R132H protein, ATRX gene mutation, p53 protein overexpression, and Ki-67 proliferation index. The following primary antibodies were used on one representative block: H09 (mouse monoclonal) clone of IDH-1 antibody (dilution 1:20; category number DIAH09, Dianova), DO7 (rabbit polyclonal) clone of ATRX antibody (dilution 1:200; category number HP A001906, sigma aldrich), Mo a Hu clone of p53 antibody (dilutions 1:100; category number M700101, Dako) and Ki-67 antibody (dilution 1:400; Cell marque). The sections were counterstained with hematoxylin and dehydrated in graded alcohol. The slides were mounted with DPX, covered with a coverslip and evaluated by an independent, experienced neuropathologist.

IDH1 R132H

Positive: Vast majority of tumor cells showed strong cytoplasmic positivity with or without nuclear staining.

Negative: No staining/ weak diffuse staining and staining of macrophages.

The mutation status of IDH1 was determined for all the cases in accordance with the current WHO classification of CNS tumors (2021). Cases were classified into: astrocytoma- IDH mutant (WHO grade 4) and glioblastoma- IDH wildtype (WHO grade 4) depending on their IDH mutation profile.

ATRX

ATRX loss: If the nuclei of tumor cells were negative (unstained) whereas the nuclei of non-neoplastic cells such as endothelia, microglia, reactive astrocytes and lymphocytes showed strong positivity. Loss of nuclear immunostaining for ATRX protein in >10% of tumor cell nuclei was considered as positive for ATRX gene mutation.

Tumor protein 53 (p53)

Overexpression: A minimum of 10% or more of strong positive tumor cell nuclei.

No overexpression: No immunostaining or staining in <10% tumor cell nuclei. 

Ki-67 proliferation indexwas defined as the average percentage of positive nuclei per 1000 nuclei at x400 magnification.

DNA extraction

Areas with high content of tumor cells (70-90%) were selected and dissected out for further analysis. Formalin-fixed paraffin embedded tissue sections were used for the extraction of Genomic DNA using standard Qiagen tissue DNA extraction kit according to the manufacturer´s protocol (Qiagen, Hilden, Germany). The extracted DNA was quantified using Nanodrop Lite Spectrophotometer, stored at appropriate temperature and used further for MS PCR.

Analysis of MGMT promoter methylation status by MS PCR

The status of MGMT promoter methylation was assessed by MS PCR. MS PCR is a method of analysing the DNA methylation patterns on CpG islands. For performing MS PCR, bisulfite-modified DNA is amplified using two pairs of primer, which detect methylated and unmethylated DNA, respectively. The protocol includes chemical modification of unmethylated cytosine to uracil, followed by a nested two-stage PCR. Steps of the procedure were as follows:

· Isolated DNA from each sample was subjected to bisufite conversion using Bisulfite conversion kit (EZ Gold DNA methylation kit; M/s. Zymo Research, Orange, CA, USA).

· Polymerase chain reaction (PCR) was performed next using methylation specific primers.

· First stage primer recognized the bisulfite modified template flanking the MGMT gene but did not discriminate between methylated and unmethylated alleles.

· Primer sequences for first stage PCR were:

5#-GGATATGTTGGGATAGTT-3# (forward primer)

5#-CCAAAAACCCCAAACCC-3# (reverse primer)

· For stage 1, the PCR amplification protocol was as follows: an initial denaturation step of 5 min at 94°C; followed by 40 cycles of 30s at 94°C, 30s at 52°C and 30s at 72°C and a final elongation step of 7 min at 72°C. Methylation and unmethylation primers were used separately for each test.

· Primer sequences for unmethylated reaction were:

5#-TTTGTGTTTTGATGTTTGTAGGTTTTTGT-3# (forward primer)

5#-AACTCCACACTCTTCCAAAAACAAAACA-3# (reverse primer)

· Primer sequences for methylated reaction were:

5#-TTTCGACGTTCGTAGGTTTTCGC-3# (forward primer)

5#-GCACTCTTCCGAAAACGAAACG-3# (reverse primer).

· For stage 2, the PCR amplification procedure was as follows: an initial denaturation step of 5 min at 94°C, followed by 35 cycles of 15s at 94°C, 15s at 62°C and 15s at 72°C and a final elongation step of 7 min at 72°C.

· MSP was performed to amplify 83 base-pair fragment of the methylated MGMT gene promoter and 91 base-pair fragment unmethylated product.

· Amplified products were then separated on 4% agarose gel, stained with ethidium bromide and visualized under UV illumination.

Statistical analysis

The data was analyzed using SPSS-24 software. Descriptive statistics of continuous variables have been presented in mean ± standard deviation / median (interquartile range) as appropriate. Categorical data has been presented in frequency (%). To compare the median distributions between two groups / three groups, independent t-test / Mann Whitney U test / Kruskal Wallis H test has been used. Chi square test has been used to compare the proportions between groups. In case any cell had an expected count < 5, Fisher’s exact test has been used in place of Chi square test. The patients were followed up for a period of three years to determine the overall survival from the day of the diagnosis. Kaplan Meier analysis was used to evaluate the survival outcome probabilities. A p value < 0.05 was considered statistically significant.

## Results

89 cases of WHO grade 4 diffuse glioma diagnosed on histopathology were included in the study. It comprised of 63 males (M) and 26 females (F) (M: F = 2.4:1). Mean age was 46.78 ± 15.49 years, and the age range was 7-73 years. Demographic and clinical details of the study cohort is presented in Table 1.

**Table 1:** Table 1. Demographic and clinical profile of the study population

Demographic and clinical characteristics		Number (n)	Percentage (%)
Age (mean + SD)	46.78±15.49		
Gender	Male	63	70.79
	Female	26	29.21
Site of tumor	Corpus callosum	3	3.37
	Frontal lobe	26	29.21
	Fronto-parietal	2	2.25
	Fronto-temporal	4	4.49
	Insula	5	5.62
	Midbrain and pons	1	1.12
	Occipetal lobe	1	1.12
	Parietal lobe	5	5.62
	Parieto-occipetal	6	6.74
	Perisylvian	2	2.25
	Temporal lobe	19	21.35
	Temporo-occipetal	2	2.25
	Temporo-parietal	5	5.62
	Thalamus	2	2.25
	Trigone	6	6.74
Clinical presentation	History of loss of consciousness	4	4.49
	Headache	68	76.40
	Vomiting	28	31.46
	Bladder/bowel incontinence	2	2.25
	Impaired vision	13	14.61
	Hemi/ Para / Quadriparesis	26	29.21
	Seizure	22	24.72
	Papilloedema	7	7.87
	Impaired memory	12	13.48
	Ataxia	25	28.09

Immunohistochemistry was applied for IDH1, ATRX, p53 and Ki-67 index in all the cases. 8/89 (9%) cases that showed positive staining for IDH1 on immunohistochemistry were designated as astrocytoma- IDH mutant (WHO grade 4) whereas 81/89 (91%) cases that were negative IDH1 immunostaining were designated as glioblastoma- IDH wildtype (WHO grade 4) in accordance with the current WHO classification of tumors of CNS (2021). Histology of both the groups was similar, showing increased cellularity with moderate to marked nuclear pleomorphism, increased mitotic activity, microvascular proliferation, and/ or necrosis. Loss in expression of ATRX protein was identified in 21.3% (19/89) cases and was more prevalent in WHO grade 4 IDH mutant astrocytomas (7/8; 87.5%) compared to IDH wildtype glioblastomas (12/81; 14.8%). p53 protein overexpression was noted in 63/89 (70.8%) cases. Among WHO grade 4 IDH mutant astrocytomas, p53 protein overexpression was noted in 87.5% (7/8 cases) whereas among IDH wildtype glioblastomas, p53 overexpression was noted in 69.1% (56/81 cases). Majority of grade 4 diffuse glioma patients had a Ki-67 proliferation index of > 10% (93.75%). The immunohistochemical profile of the patient has been presented in Table 2.

**Table 2:** Table 2. Immunohistochemical profile of the study population

IHC markers		Number (n)	%
IDH	Negative	81	91.01
	Positive	8	8.99
p53	No overexpression	26	29.21
	Overexpression	63	70.79
ATRX	Loss	40	44.94
	Retained	49	55.06
Ki-67	<10%	2	2.25
	>10%	87	97.75

**Figure 1:**
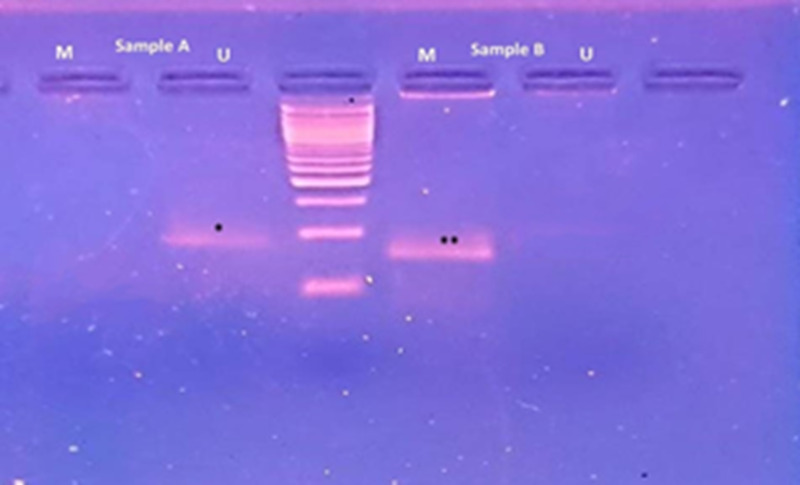
Figure 1. Gel electrophoresis of nested PCR products. **Sample A:** displaying unmethylated **(U)** MGMT promoter (91 base-pair fragment) [indicated by *] and **Sample** **B: **displaying methylated (M) MGMT promoter (83 base-pair fragment) [indicated by **]. The ladder is of 50 base pairs.

Methylation of MGMT promoter was detected in 38.2% (34/89) cases. The group comprising of IDH-mutant WHO grade 4 astrocytomas frequently showed methylation of MGMT promoter (6/8; 75%). Among glioblastomas- IDH wildtype, methylation of MGMT promoter was observed in 34.6% (28/81) cases. This difference in the methylation profile among two groups was statistically significant (p = 0.05). A representative photograph of methylated MGMT promoter is shown in Figure 1.

Among cases with methylated MGMT promoter, 76.5% were p53 positive (26/34) while among cases with unmethylated MGMT promoter, 67.3% showed p53 positivity (37/55 cases). This difference was not statistically significant (p = 0.492) (Table 3). There was no significant statistical difference in the Ki-67 proliferation indices between the cases with methylated and unmethylated MGMT promoter (p = 0.698) (Table 3).

The median overall survival curves for WHO grade 4 diffuse glioma patients receiving standard radiation therapy and concomitant temozolomide chemotherapy showed a trend towards better survival in group with methylated MGMT promoter [29.73 months (SE 1.11)] compared to the group with unmethylated MGMT promoter [10.20 months (SE 0.70)].

**Figure 2:**
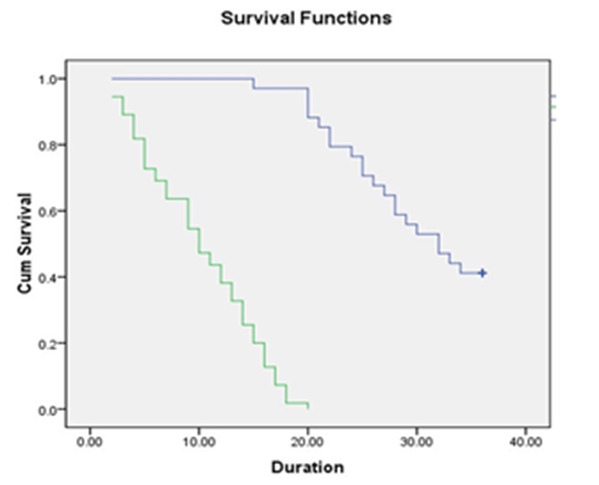
Figure 2. Kaplan Meier curve showing survival outcomes in patients with methylated (presented by blue line) and unmethylated (presented by green line) MGMT tumors

This difference was statistically significant (p < 0.001). This improved survival in patients with methylated MGMT promoter occurred exclusively at 36 months of follow-up. Also, compared to patients with methylated MGMT promoter, patients with unmethylated MGMT had an increased risk of death at 6 months of follow up. Methylated MGMT promoter was significantly associated with progressively longer survival (Figure 2).

**Table 3:** Table 3. The clinicopathological profile of MGMT promoter methylated and unmethylated tumors

Patient characteristics		MGMT promoter methylation				
		Methylated (n=34)		Unmethylated (n=55)		
		N	%	N	%	p-value
Age (years)	<18	4	11.76	2	3.64	0.527
	18-30	4	11.76	4	7.27	
	31-40	6	17.65	7	12.73	
	41-50	6	17.65	14	25.45	
	51-60	9	26.47	17	30.91	
	>60	5	14.71	12	21.82	
Gender	Male	25	73.53	38	69.09	0.836
	Female	9	26.47	17	30.91	
Mental Status	Normal	29	85.29	51	92.73	0.442
	Impaired	5	14.71	4	7.27	
Site of tumor	Corpus callosum	2	5.88	5	5.45	0.788
	Frontal lobe	12	35.29	14	25.45	0.346
	Insula	2	5.88	5	9.09	0.788
	Occipetal lobe	0	0.00	2	3.63	0.888
	Parietal lobe	3	8.82	2	3.64	0.366
	Parieto-occipetal	2	5.88	4	7.27	1.00
	Temporal lobe	7	20.59	12	21.82	1.00
	Temporo-parietal	3	8.82	3	5.45	0.366
	Thalamus	1	2.94	3	5.45	0.810
	Trigone	2	5.88	5	9.09	0.788
Clinical signs and symptoms	History of loss of consciousness	1	2.94	3	5.45	0.976
	Headache	24	70.59	44	80.00	0.448
	Vomiting	12	35.29	16	29.09	0.706
	Bladder/bowel incontinence	1	2.94	1	1.82	0.728
	Impaired vision	4	11.76	9	16.36	0.773
	Hemi/ Para / Quadriparesis	14	41.18	12	21.82	0.087
	Seizure	6	17.65	16	29.09	0.336
	Papilledema	2	5.88	5	9.09	0.888
	Impaired memory	2	5.88	10	18.18	0.183
	Ataxia	9	26.47	16	29.09	0.980
IDH1 R132H (8/89)	Mutation present	6	17.65	2	3.64	0.050
p53 (63/89	Overexpression	26	76.47	37	67.27	0.492
ATRX (19/89)	Loss of ATRX	14	41.2	5	9.1	0.003
Ki-67	<10%	0	0.00	2	3.64	0.698
	>10%	34	100.00	53	96.36	
Survival Status	Alive	14	41.18	0	0.00	<0.001
	Death	20	58.82	55	100.00	

## Discussion

CNS gliomas are the commonest primary malignant brain tumor and comprise multiple types in particular astrocytoma, oligodendroglioma, ependymoma and glioblastoma. IDH-wildtype glioblastoma represents the most frequent glioma type with aggressive behaviour and poorer outcome. It has the propensity for widespread invasion including invasion to adjacent tissues^18^. The average median survival of patients with glioblastoma is very less even after aggressive treatment^19,20^. Also, prognosis of the patients with glioblastoma has remained extremely poor despite multimodal treatment approaches including surgery, radiotherapy and chemotherapy^19^. Methylation of MGMT promoter gene followed by subsequent inactivation of its protein has been substantiated to modulate the response of chemotherapeutic drugs such as temozolomide (an alkylating agent), commonly used in malignant gliomas^18^. Few studies in in literature have authenticated that methylation of MGMT promoter sequence serves as a powerful prognostic and predictive tool for longer overall survival and progression free survival in patients with glioblastoma ^1,13,21^.

The frequency rate of methylated MGMT promoter in glioma patients has shown discrepancies across the globe. In literature, the frequency of MGMT promoter methylation by MS PCR varies from as low as 35% to as high as 68%^12,22-28^. In the present study, frequency of MGMT promoter methylation by MS PCR among WHO grade 4 diffuse glioma patients was recorded to be 38.2%. A comparison of the frequency of MGMT promoter methylation in present study with the existing literature has been shown in Table 4.

In the current study, no significant difference was observed among the methylated and unmethylated tumors in terms of age (p = 0.527) and sex (p = 0.836). Similar results were noted by Arora I et al^19^ in their study on 134 glioblastoma patients. Frontal lobe (35.3%) was the preferred location for tumors with both methylated and unmethylated MGMT promoter.This may be due to the fact that frontal lobe being the largest is also the commonest site for gliomas. This association, however, was not statistically significant (p = 0.548). Similar observations were made by Wang Y et al^^29^^.

In literature, the association between p53 overexpression and methylated MGMT promoter is highly contradictory. Shamshara J et al^30^ observed a statistically significant association between p53 mutations and methylated MGMT promoter while Jesien-Lewandowicz E et al^31^andJha P et al^32^did not find any association between the two. Current study did not show a significant association between p53 overexpression and methylated MGMT promoter.

Arora I et al^19^ observed a statistically significant association between methylated MGMT promoter and IDH1 mutation as well as loss of ATRX protein expression, whereas Jha P et al^32^did not find any association between the two. There was a statistically significant association of methylated MGMT promoter tumors with IDH1 mutation (p = 0.050) and loss of ATRX protein expression (p = 0.003) in the present study. The tumors with methylated MGMT promoter status showed greater frequencies of IDH1 protein expression and loss of ATRX gene mutation compared to the unmethylated tumors.

IIn an EORTC/NCIC phase III clinical trial^6,33^ was observed that the patients with MGMT methylated tumors live longer, irrespective of the treatment. In their study, the median overall survival of patients with methylated MGMT promoter was 18.2 months compared to only 12.2 months in patients without MGMT promoter methylation. In the same study, a 5‑year survival analysis demonstrated MGMT promoter methylation as the strongest predictor of treatment outcome and benefit from alkylating agent chemotherapy. The median overall survival was 29.7 months among patients with methylated MGMT promoter whereas it was only 10.2 months among patients with unmethylated MGMT promoter in our cohort of 89 WHO grade 4 diffuse glioma patients treated with standard of care radiation therapy and temozolomide chemotherapy. First-line single chemotherapeutic agent ‘temozolomide’ was associated with significantly improved overall survival in these patients. The findings of our results showed that temozolomide is an effective therapy for all WHO grade 4 glioma patients irrespective of their IDH mutation profile and methylated MGMT promoter is a good prognostic marker in the setting of treatment with an alkylating agent (temozolomide); and may be predictive of improved overall survival.

**Table 4:** Table 4. A comparison of the frequency of MGMT promoter methylation in present study with the existing literature

S. No.	Author	Number of cases analysed	MGMT promoter methylation (%)
1.	Jovanovic N et al. Serbia (2019)^34^	25 glioblastoma	12/25 (48%)
2.	Johanessen E et al. Oslo University Hospital, Montebello, Norway (2018)^35^	48 glioblastoma	23/48 (47.9%)
3.	Arora I et al. TMH, Mumbai, India (2018)^19^	134 glioblastoma	66/134 (49.1%)
4.	Uno M et al. Ludwig Institute for Cancer Research, Brazil (2011)^36^	51 glioblastoma	22/51 (43.1%)
5.	Jovanovic N et al. Serbia (2019)^34^	25 glioblastoma	12/25 (48%)
6.	Nehru GA et al. CMC, Vellore, India (2012)^20^	27 glioblastoma	17/27 (62%)
7.	Brandes AA et al. Italy (2008)^27^	103 glioblastoma	36/103 (35%)
8.	Eoli M et al. Milan, Italy (2007)^25^	86 glioblastoma	41/86 (47.7%)
9.	Present study	89 WHO grade 4 diffuse gliomas	34/89 (38.2%) IDH-mutant WHO grade 4 astrocytoma- 6/8(75%) IDH- wildtype glioblastoma - 28/81 (34.6%)

## Conclusion

Our study suggests that methylation of MGMT promoter sequence is seen in greater proportion in WHO grade 4 diffuse gliomas that also express IDH1 immunopositivity and loss of ATRX protein expression. Also, methylation of MGMT promoter is an important prognostic and predictive biomarker of better treatment outcome and is significantly associated with longer overall survival in grade 4 diffuse glioma cases receiving radiation therapy and concomitant alkylating agent chemotherapy. First-line single chemotherapeutic agent ‘temozolomide’ has been associated with significantly improved survival in these patients if MGMT promoter is methylated. Thus, for a clinician, it is important to confirm the methylation status of MGMT before starting the chemotherapeutic treatment of grade 4 diffuse glioma patients after surgery.

## References

[1] Stupp Roger, Mason Warren P., van den Bent Martin J., Weller Michael, Fisher Barbara, Taphoorn Martin J.B., Belanger Karl, Brandes Alba A., Marosi Christine, Bogdahn Ulrich, Curschmann Jürgen, Janzer Robert C., Ludwin Samuel K., Gorlia Thierry, Allgeier Anouk, Lacombe Denis, Cairncross J. Gregory, Eisenhauer Elizabeth, Mirimanoff René O. (2005). Radiotherapy plus Concomitant and Adjuvant Temozolomide for Glioblastoma. New England Journal of Medicine.

[2] Louis David N., Perry Arie, Reifenberger Guido, von Deimling Andreas, Figarella-Branger Dominique, Cavenee Webster K., Ohgaki Hiroko, Wiestler Otmar D., Kleihues Paul, Ellison David W. (2016). The 2016 World Health Organization Classification of Tumors of the Central Nervous System: a summary. Acta Neuropathologica.

[3] Stupp Roger, Taillibert Sophie, Kanner Andrew A., Kesari Santosh, Steinberg David M., Toms Steven A., Taylor Lynne P., Lieberman Frank, Silvani Antonio, Fink Karen L., Barnett Gene H., Zhu Jay-Jiguang, Henson John W., Engelhard Herbert H., Chen Thomas C., Tran David D., Sroubek Jan, Tran Nam D., Hottinger Andreas F., Landolfi Joseph, Desai Rajiv, Caroli Manuela, Kew Yvonne, Honnorat Jerome, Idbaih Ahmed, Kirson Eilon D., Weinberg Uri, Palti Yoram, Hegi Monika E., Ram Zvi (2015). Maintenance Therapy With Tumor-Treating Fields Plus Temozolomide vs Temozolomide Alone for Glioblastoma. JAMA.

[4] Parker Nicole R, Hudson Amanda L, Khong Peter, Parkinson Jonathon F, Dwight Trisha, Ikin Rowan J, Zhu Ying, Cheng Zhangkai Jason, Vafaee Fatemeh, Chen Jason, Wheeler Helen R, Howell Viive M (2016). Intratumoral heterogeneity identified at the epigenetic, genetic and transcriptional level in glioblastoma.. Scientific reports.

[5] Zhang Jihong, F.G. Stevens Malcolm, D. Bradshaw Tracey (2012). Temozolomide: Mechanisms of Action, Repair and Resistance. Current Molecular Pharmacology.

[6] Hegi Monika E, Liu Lili, Herman James G, Stupp Roger, Wick Wolfgang, Weller Michael, Mehta Minesh P, Gilbert Mark R (2008). Correlation of O6-methylguanine methyltransferase (MGMT) promoter methylation with clinical outcomes in glioblastoma and clinical strategies to modulate MGMT activity.. Journal of clinical oncology : official journal of the American Society of Clinical Oncology.

[7] Sciuscio Davide, Diserens Annie-Claire, van Dommelen Kristof, Martinet Danielle, Jones Greg, Janzer Robert-Charles, Pollo Claudio, Hamou Marie-France, Kaina Bernd, Stupp Roger, Levivier Marc, Hegi Monika E (2011). Extent and patterns of MGMT promoter methylation in glioblastoma- and respective glioblastoma-derived spheres.. Clinical cancer research : an official journal of the American Association for Cancer Research.

[8] Gerson Stanton L (2002). Clinical relevance of MGMT in the treatment of cancer.. Journal of clinical oncology : official journal of the American Society of Clinical Oncology.

[9] Esteller M, Garcia-Foncillas J, Andion E, Goodman S N, Hidalgo O F, Vanaclocha V, Baylin S B, Herman J G (2000). Inactivation of the DNA-repair gene MGMT and the clinical response of gliomas to alkylating agents.. The New England journal of medicine.

[10] Bobola M S, Berger M S, Ellenbogen R G, Roberts T S, Geyer J R, Silber J R (2001). O6-Methylguanine-DNA methyltransferase in pediatric primary brain tumors: relation to patient and tumor characteristics.. Clinical cancer research : an official journal of the American Association for Cancer Research.

[11] Cabrini Giulio, Fabbri Enrica, Lo Nigro Cristiana, Dechecchi Maria Cristina, Gambari Roberto (2015). Regulation of expression of O6-methylguanine-DNA methyltransferase and the treatment of glioblastoma (Review).. International journal of oncology.

[12] Hegi Monika E, Diserens Annie-Claire, Gorlia Thierry, Hamou Marie-France, de Tribolet Nicolas, Weller Michael, Kros Johan M, Hainfellner Johannes A, Mason Warren, Mariani Luigi, Bromberg Jacoline E C, Hau Peter, Mirimanoff René O, Cairncross J Gregory, Janzer Robert C, Stupp Roger (2005). MGMT gene silencing and benefit from temozolomide in glioblastoma.. The New England journal of medicine.

[13] Weller Michael, Stupp Roger, Reifenberger Guido, Brandes Alba A, van den Bent Martin J, Wick Wolfgang, Hegi Monika E (2010). MGMT promoter methylation in malignant gliomas: ready for personalized medicine?. Nature reviews. Neurology.

[14] Herman J G, Graff J R, Myöhänen S, Nelkin B D, Baylin S B (1996). Methylation-specific PCR: a novel PCR assay for methylation status of CpG islands.. Proceedings of the National Academy of Sciences of the United States of America.

[15] Friedman H S, McLendon R E, Kerby T, Dugan M, Bigner S H, Henry A J, Ashley D M, Krischer J, Lovell S, Rasheed K, Marchev F, Seman A J, Cokgor I, Rich J, Stewart E, Colvin O M, Provenzale J M, Bigner D D, Haglund M M, Friedman A H, Modrich P L (1998). DNA mismatch repair and O6-alkylguanine-DNA alkyltransferase analysis and response to Temodal in newly diagnosed malignant glioma.. Journal of clinical oncology : official journal of the American Society of Clinical Oncology.

[16] Shilpa V, Bhagat Rahul, Premalata C S, Pallavi V R, Ramesh G, Krishnamoorthy Lakshmi (2014). Relationship between promoter methylation & tissue expression of MGMT gene in ovarian cancer.. The Indian journal of medical research.

[17] Felsberg Jörg, Rapp Marion, Loeser Simon, Fimmers Rolf, Stummer Walter, Goeppert Matthias, Steiger Hans-Jacob, Friedensdorf Britta, Reifenberger Guido, Sabel Michael C (2009). Prognostic significance of molecular markers and extent of resection in primary glioblastoma patients.. Clinical cancer research : an official journal of the American Association for Cancer Research.

[18] Pandith Arshad A, Qasim Iqbal, Zahoor Wani, Shah Parveen, Bhat Abdul R, Sanadhya Dheera, Shah Zafar A, Naikoo Niyaz A (2018). Concordant association validates MGMT methylation and protein expression as favorable prognostic factors in glioma patients on alkylating chemotherapy (Temozolomide).. Scientific reports.

[19] Arora Iteeka, Gurav Mamta, Rumde Rachna, Dhanavade Sandeep, Kadam Vinayak, Kurani Hetakshi, Shetty Omshree, Goda Jayant Sastri, Shetty Prakash, Moiyadi Aliasgar, Gupta Tejpal, Jalali Rakesh, Epari Sridhar (2018). MGMT gene promoter methylation and its correlation with clinicopathological parameters in glioblastomas.. Neurology India.

[20] Nehru Gopal Arun, Pai Rekha, Samuel Prasanna, Chacko Ari G., Chacko Geeta (2012). Status of O 6 -methylguanine-DNA methyltransferase [MGMT] gene promoter methylation among patients with glioblastomas from India. Neurology India.

[21] Srivastava Arti, Jain Ayushi, Jha Prerana, Suri Vaishali, Sharma Mehar Chand, Mallick Supriya, Puri Tarun, Gupta Deepak Kumar, Gupta Aditya, Sarkar Chitra (2010). MGMT gene promoter methylation in pediatric glioblastomas.. Child's nervous system : ChNS : official journal of the International Society for Pediatric Neurosurgery.

[22] Weller Michael, Felsberg Jörg, Hartmann Christian, Berger Hilmar, Steinbach Joachim P, Schramm Johannes, Westphal Manfred, Schackert Gabriele, Simon Matthias, Tonn Jörg C, Heese Oliver, Krex Dietmar, Nikkhah Guido, Pietsch Torsten, Wiestler Otmar, Reifenberger Guido, von Deimling Andreas, Loeffler Markus (2009). Molecular predictors of progression-free and overall survival in patients with newly diagnosed glioblastoma: a prospective translational study of the German Glioma Network.. Journal of clinical oncology : official journal of the American Society of Clinical Oncology.

[23] Herrlinger Ulrich, Rieger Johannes, Koch Dorothee, Loeser Simon, Blaschke Britta, Kortmann Rolf-Dieter, Steinbach Joachim P, Hundsberger Thomas, Wick Wolfgang, Meyermann Richard, Tan Ta-Chih, Sommer Clemens, Bamberg Michael, Reifenberger Guido, Weller Michael (2006). Phase II trial of lomustine plus temozolomide chemotherapy in addition to radiotherapy in newly diagnosed glioblastoma: UKT-03.. Journal of clinical oncology : official journal of the American Society of Clinical Oncology.

[24] Hegi Monika E, Diserens Annie-Claire, Godard Sophie, Dietrich Pierre-Yves, Regli Luca, Ostermann Sandrine, Otten Philippe, Van Melle Guy, de Tribolet Nicolas, Stupp Roger (2004). Clinical trial substantiates the predictive value of O-6-methylguanine-DNA methyltransferase promoter methylation in glioblastoma patients treated with temozolomide.. Clinical cancer research : an official journal of the American Association for Cancer Research.

[25] Eoli Marica, Menghi Francesca, Bruzzone Maria Grazia, De Simone Tiziana, Valletta Lorella, Pollo Bianca, Bissola Lorena, Silvani Antonio, Bianchessi Donatella, D'Incerti Ludovico, Filippini Graziella, Broggi Giovanni, Boiardi Amerigo, Finocchiaro Gaetano (2007). Methylation of O6-methylguanine DNA methyltransferase and loss of heterozygosity on 19q and/or 17p are overlapping features of secondary glioblastomas with prolonged survival.. Clinical cancer research : an official journal of the American Association for Cancer Research.

[26] Li Yucai, Shan Xia, Wu Zhifeng, Wang Yinyan, Ling Miao, Fan Xing (2018). IDH1 mutation is associated with a higher preoperative seizure incidence in low-grade glioma: A systematic review and meta-analysis.. Seizure.

[27] Brandes Alba A, Franceschi Enrico, Tosoni Alicia, Blatt Valeria, Pession Annalisa, Tallini Giovanni, Bertorelle Roberta, Bartolini Stefania, Calbucci Fabio, Andreoli Alvaro, Frezza Giampiero, Leonardi Marco, Spagnolli Federica, Ermani Mario (2008). MGMT promoter methylation status can predict the incidence and outcome of pseudoprogression after concomitant radiochemotherapy in newly diagnosed glioblastoma patients.. Journal of clinical oncology : official journal of the American Society of Clinical Oncology.

[28] Wick Wolfgang, Stupp Roger, Beule Anna-Carina, Bromberg Jacoline, Wick Antje, Ernemann Ulrike, Platten Michael, Marosi Christine, Mason Warren P, van den Bent Martin, Weller Michael, Rorden Chris, Karnath Hans-Otto (2008). A novel tool to analyze MRI recurrence patterns in glioblastoma.. Neuro-oncology.

[29] Wang Yinyan, Fan Xing, Zhang Chuanbao, Zhang Tan, Peng Xiaoxia, Li Shaowu, Wang Lei, Ma Jun, Jiang Tao (2014). Anatomical specificity of O6-methylguanine DNA methyltransferase protein expression in glioblastomas.. Journal of neuro-oncology.

[30] Shamsara Jamal, Sharif Samaneh, Afsharnezhad Sima, Lotfi Marzieh, Raziee Hamid Reza, Ghaffarzadegan Kamran, Moradi Afshin, Rahighi Saeed, Behravan Javad (2009). Association between MGMT promoter hypermethylation and p53 mutation in glioblastoma.. Cancer investigation.

[31] Jesien-Lewandowicz Emilia, Jesionek-Kupnicka Dorota, Zawlik Izabela, Szybka Małgorzata, Kulczycka-Wojdala Dominika, Rieske Piotr, Sieruta Monika, Jaskolski Dariusz, Och Waldemar, Skowronski Wiesław, Sikorska Beata, Potemski Piotr, Papierz Wielislaw, Liberski Pawel P, Kordek Radzisław (2009). High incidence of MGMT promoter methylation in primary glioblastomas without correlation with TP53 gene mutations.. Cancer genetics and cytogenetics.

[32] Jha Prerana, Suri Vaishali, Jain Ayushi, Sharma Mehar Chand, Pathak Pankaj, Jha Pankaj, Srivastava Arti, Suri Ashish, Gupta Deepak, Chosdol Kunzang, Chattopadhyay Parthoprasad, Sarkar Chitra (2010). O6-methylguanine DNA methyltransferase gene promoter methylation status in gliomas and its correlation with other molecular alterations: first Indian report with review of challenges for use in customized treatment.. Neurosurgery.

[33] Stupp R., Hegi M.E., Mason W.P,, van den Bent M.J., European Organisation for Research and Treatment of Cancer Brain Tumour and Radiation Oncology Groups, National Cancer Institute of Canada Clinical Trials Group (2009). Effects of radiotherapy with concomitant and adjuvant temozolomide versus radiotherapy alone on survival in glioblastoma in a randomised phase III study: 5-year analysis of the EORTC-NCIC trial. Lancet Oncol..

[34] Jovanović Nikola, Mitrović Tatjana, Cvetković Vladimir J, Tošić Svetlana, Vitorović Jelena, Stamenković Slaviša, Nikolov Vesna, Kostić Aleksandar, Vidović Nataša, Krstić Miljan, Jevtović-Stoimenov Tatjana, Pavlović Dušica (2019). The Impact of MGMT Promoter Methylation and Temozolomide Treatment in Serbian Patients with Primary Glioblastoma.. Medicina (Kaunas, Lithuania).

[35] Johannessen Lene E, Brandal Petter, Myklebust Tor Åge, Heim Sverre, Micci Francesca, Panagopoulos Ioannis (2018). MGMT Gene Promoter Methylation Status - Assessment of Two Pyrosequencing Kits and Three Methylation-specific PCR Methods for their Predictive Capacity in Glioblastomas.. Cancer genomics & proteomics.

[36] Uno Miyuki, Oba-Shinjo Sueli Mieko, Camargo Anamaria Aranha, Moura Ricardo Pereira, Aguiar Paulo Henrique de, Cabrera Hector Navarro, Begnami Marcos, Rosemberg Sérgio, Teixeira Manoel Jacobsen, Marie Suely Kazue Nagahashi (2011). Correlation of MGMT promoter methylation status with gene and protein expression levels in glioblastoma.. Clinics (Sao Paulo, Brazil).

